# Autophagy Creates a CTL Epitope That Mimics Tumor-Associated Antigens

**DOI:** 10.1371/journal.pone.0047126

**Published:** 2012-10-11

**Authors:** Ayako Demachi-Okamura, Hiroki Torikai, Yoshiki Akatsuka, Hiroyuki Miyoshi, Tamotsu Yoshimori, Kiyotaka Kuzushima

**Affiliations:** 1 Division of Immunology, Aichi Cancer Center Research Institute, Nagoya, Japan; 2 Department of Immunology, The University of Texas MD Anderson Cancer Center, Houston, Texas, United States of America; 3 Department of Hematology and Oncology, Fujita Health University, Toyoake, Japan; 4 Subteam for Manipulation of Cell Fate, RIKEN BioResource Center, Tsukuba, Japan; 5 Department of Genetics, Graduate School of Medicine, Osaka University, Osaka, Japan; 6 Department of Cellular Oncology, Nagoya University Graduate School of Medicine, Nagoya, Japan; Mie University Graduate School of Medicine, United States of America

## Abstract

The detailed mechanisms responsible for processing tumor-associated antigens and presenting them to CTLs remain to be fully elucidated. In this study, we demonstrate a unique CTL epitope generated from the ubiquitous protein puromycin-sensitive aminopeptidase, which is presented via HLA-A24 on leukemic and pancreatic cancer cells but not on normal fibroblasts or EBV-transformed B lymphoblastoid cells. The generation of this epitope requires proteasomal digestion and transportation from the endoplasmic reticulum to the Golgi apparatus and is sensitive to chloroquine-induced inhibition of acidification inside the endosome/lysosome. Epitope liberation depends on constitutively active autophagy, as confirmed with immunocytochemistry for the autophagosome marker LC3 as well as RNA interference targeting two different autophagy-related genes. Therefore, ubiquitously expressed proteins may be sources of specific tumor-associated antigens when processed through a unique mechanism involving autophagy.

## Introduction

Exploring the mechanisms underlying cancer-specific CTL recognition is important in the establishment of safe and effective immunotherapy. The discrimination of normal and malignant cells by CTLs depends on the repertoire of antigenic peptides displayed via the MHC class I molecules of these cells. As both normal and malignant cells possess antigen-processing machinery, the repertoire displayed depends on the expression level of the target proteins. These target proteins are degraded in the cytoplasm by the proteasome, with the resulting short peptides being translocated into the endoplasmic reticulum, where they bind to MHC class I molecules [1]. Therefore, tumor antigens are bascially determined by their expression pattern, not by the machinery responsible for the processing. If malignant cells possess unique antigen-processing machinery, they may create cancer-specific antigenic peptides, even from ubiquitously expressed proteins.

Autophagy is equally as important for peptide degradation as is proteasomal lysis of cytoplasmic proteins and organelles [2]. In macroautophagy (referred to as autophagy hereafter), autophagosomes are formed and then fused with lysosomes to produce autolysosomes, where proteins are degraded by lysosomal proteases. Upregulation of autophagy is an adaptation to stresses such as starvation, oxidant injury and genomic damage. Although autophagy normally functions under physiological conditions, degradation by the autophagosome is also important under aberrant conditions, such as cancer [3]. Thus, autophagy plays a pivotal role not only in suppressing tumorigenesis but also in promoting tumor progression. Autophagy is also known to affect both innate and adaptive immunity [4], [5]. Particularly in the latter, autophagy participates in MHC class II antigen presentation [6], [7], although reports regarding MHC class I presentation via autophagy are sparse.

Herein, we provide evidence of a cancer-specific CTL epitope created through both autophagy and proteasomal action, derived from the ubiquitously expressed protein puromycin-sensitive aminopeptidase (PSA). The data suggest that unique processing accounts for differential epitope liberation between normal and cancer cells.

## Results

### Artificial Antigen Presenting Cells (aAPCs) Effectively Induce Tumor-specific CTLs

**Figure 1:**
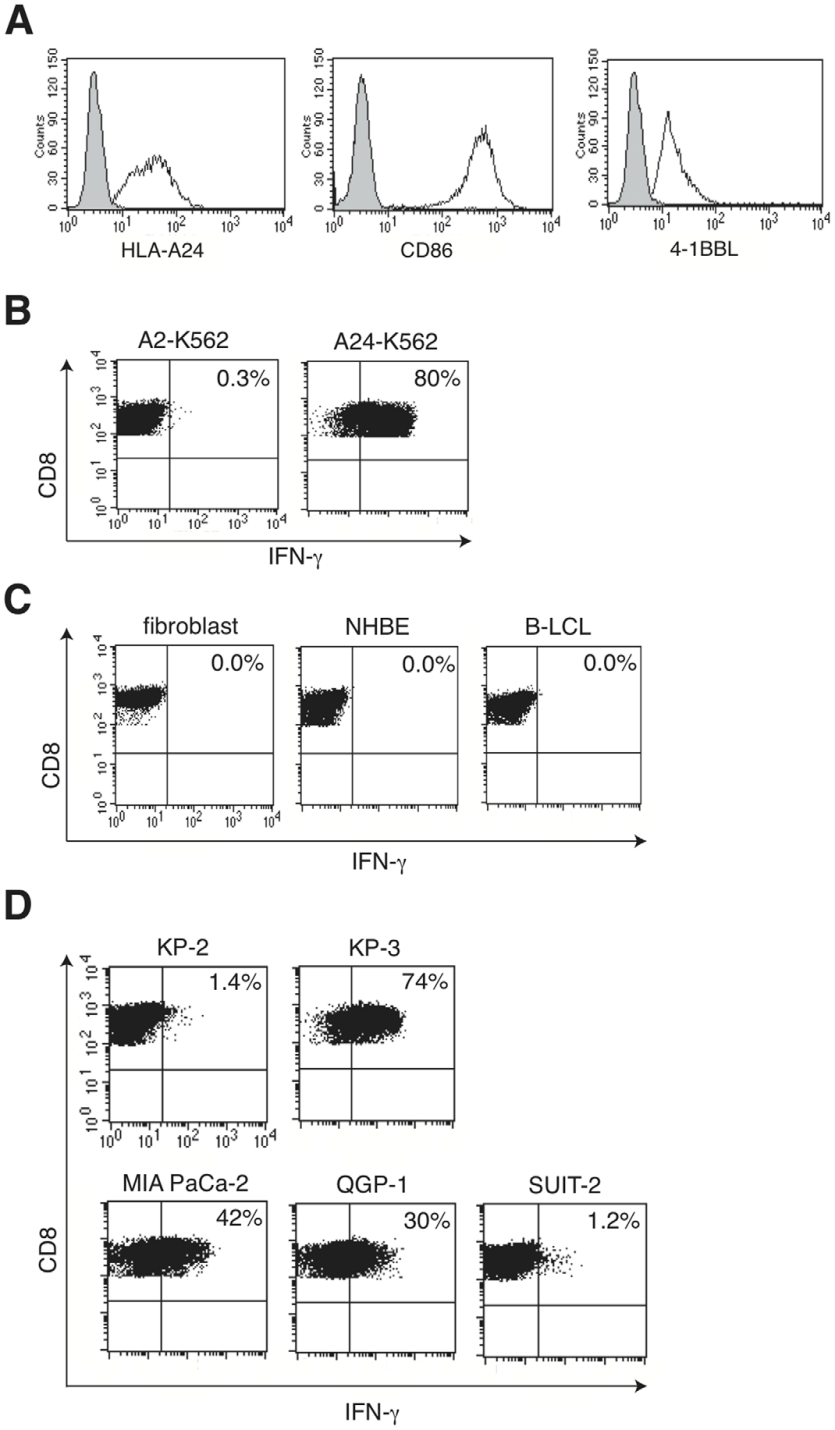
Characterization of a CTL clone, designated as 16F3, from in vitro culture with aAPCs. A, Surface expression of HLA-A24, CD86 and 4-1BBL molecules on the K562 cells used for stimulation. The shaded and solid area show non-transduced and lentiviral-transduced cells, respectively. B–D, IFN-γ secretion of 16F3 upon incubation with various cells. The 16F3 cells were incubated with K562 cells expressing either HLA-A2 or HLA-A24 (B) for 4 h, and the IFN-γ secreting cells were detected and analyzed. The frequency is shown as the percentage of the total living CD8^+^ T cells. HLA-A24-positive fibroblast cells, normal human bronchial epithelial cells and B-LCLs were used as representative non-cancerous cells (C). Five HLA-A24-positive pancreatic carcinoma cell lines were also used as stimulator cells (D). The data are representative of three independent experiments.

Generating tumor-specific CTLs generally requires autologous tumor cell lines. To bypass the substantial difficulties in establishing such lines, we have sought to use aAPCs to express endogenous tumor-associated peptides on given HLA molecules. The K562 cell line is an ideal platform for this use due to the absence of HLA expression on their cell surface; thus, K562 cells could serve as APCs when an HLA needs to be exogenously expressed [8]. To establish the K562-based aAPCs, K562 cells were stably transduced with HLA-A24, CD86 and 4-1BBL with lentiviral vectors, and a positive population was isolated (Figure 1A) to generate tumor-specific T cells from HLA-A24-positive donors. After 2 rounds of stimulation, polyclonal T cells secreted IFN-γ in an antigen-specific manner. Using a limiting dilution culture of the bulk CTL line, we established a T-cell clone, designated as 16F3, that produced IFN-γ against HLA-A24-expressing K562 cells (referred to as A24-K562) but not against dermal fibroblast cells, normal bronchial epithelial cells or EBV-transformed B lymphocytes (B-LCLs) (Figure 1B, 1C). Moreover, 16F3 recognized three out of five pancreatic cancer cell lines (Figure 1D) in an effect that was blocked by anti-HLA class I Abs (data not shown). These cell lines were positive for HLA-A24 expression (data not shown). However, 16F3 did not produce IFN-γ in response to T2-A24 cells pulsed with the 22 HLA-A24-restricted peptides derived from previously reported tumor antignes (data not shown).

### PSA-derived Epitope Induces a Specific CTL

As 16F3 recognized antigens specifically expressed on cancer cells (Figure 1B–D), we explored its nature with a cDNA library derived from K562 cellular mRNA. The library was cloned into an expression vector and divided into 960 pools, each containing 100 cDNA clones. Plasmid DNA was extracted from each pool and then transfected into HLA-A24-expressing HEK-293T (referred to as A24-293T) cells. In the first screening, one of the pools induced 16F3 to produce IFN-γ (data not shown). The positive pool was subcloned into individual cDNA clones and screened (Figure S1A). The single clone, 8G, that induced 16F3 to produce IFN-γ proved to be a variant cDNA of PSA (NM_006310). This variant PSA cDNA is generated via intronic polyadenylation [9] and consists of exons 1–12 with part of an intron located directly downstream of exon 12 (Figure S1B). To identify the epitope recognized by 16F3, A24-293T cells were transfected with plasmids encoding truncated forms of PSA. Upon analysis, the antigenicity disappeared with the following mutations: C-terminal truncation between amino acid residues 200 and 300 and N-terminal truncation between 241 and 251 (Figure S1C). To define the N- and C-terminal ends more precisely, further truncation was performed within this region. As inclusion of isoleucine at position 261 was essential for the stimulation of the clone (Figure S1D), this amino acid was indicated to be located in the C-terminus. Exclusion of aspartic acid at position 250 abolished antigenicity; thus, we considered an unusually long 12–mer peptide, DYFNVPYPLPKI (residues 250–261), having tyrosine at the second position as a primary anchor for HLA-A24 binding [10], to be the minimal epitope. A synthesized 12-mer peptide (residue 250–261) was demonstrated to bind HLA-A24 molecules more efficiently than a CMV pp65 peptide (Figure S1E **)** and to be recognized by 16F3 (Figure S1F). Failure of recognition of KP-2 and SUIT-2 cells by 16F3 was attributable to the absence of epitope presentation because both are recognized by 16F3 when pulsed with the peptide (Figure S2). A 9–mer peptide, DYFNVPYPL (residue 250–258), which was highly scored for HLA-A24 binding by the BIMAS software (http://www-bimas.cit.nih.gov/molbio/hla_bind/), was not recognized by 16F3 (Figure S1D).

### PSA has Two Variant Forms to be Presented

**Figure 2:**
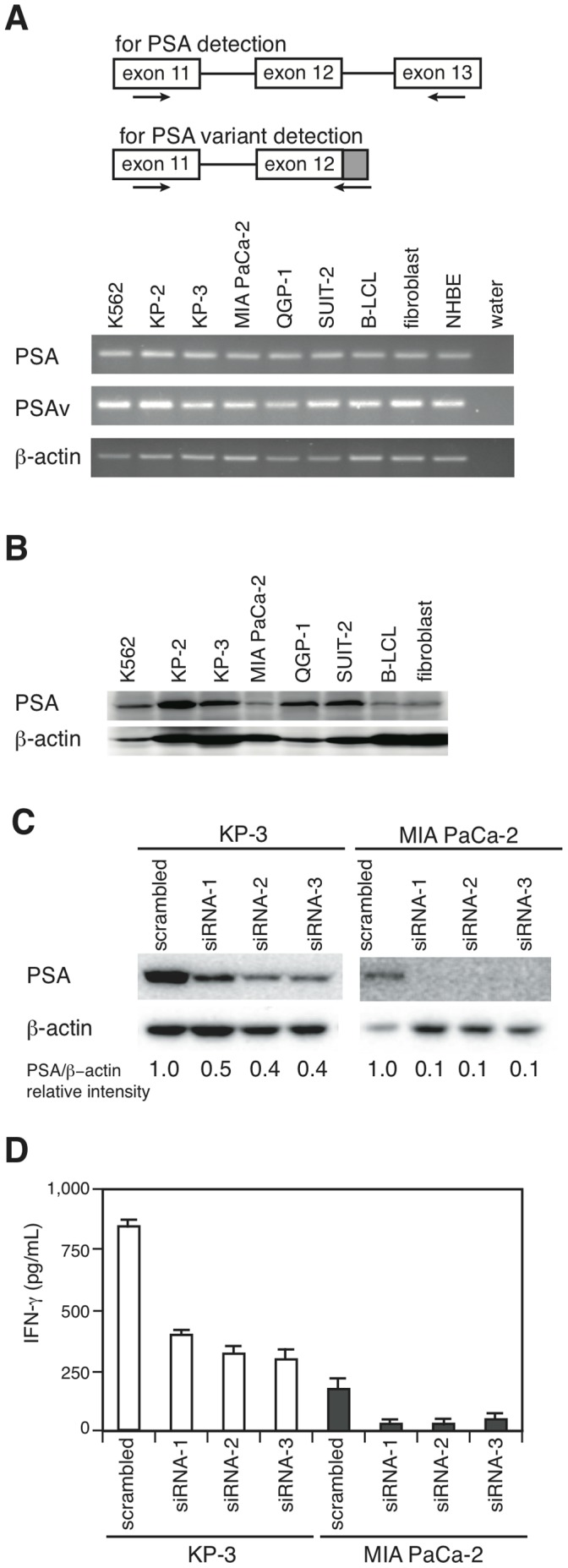
Ubiquitous expression of the PSA mRNA and protein within various cells irrespective of clone recognition. A, Two RT-PCR primer sets are designed to discriminate between wild type and variant PSA (PSAv), indicated by horizontal arrows (top). RT-PCR analysis was performed using the PSA primer sets and those for β-actin (bottom). B, The expression of the PSA and β-actin proteins was analyzed using Western blot analysis. C, KP-3 and MIA PaCa-2 cells were transfected for 70 h with scrambled or PSA-specific siRNA. Three different siRNA targets were chosen for the PSA gene, and Western blot analysis of the PSA protein in siRNA-treated cells was performed. The intensities of the bands were calculated using the ImageJ software. D, CTL responses against siRNA-treated cells were examined using an IFN-γ ELISA. The results are expressed as the means ± SD of triplicate values. Similar results were obtained in three separate experiments.

The PSA protein is expressed ubiquitously [11]. Thus, we investigated the expression levels of both full-length and variant PSA mRNA with RT-PCR. The primers for detecting the full-length forms corresponded to exon 11 and exon 13, and those for the variant forms corresponded to exon 11 and part of the intron located directly downstream of exon 12 (Figure 2A, top). Both forms of PSA mRNA were detected in all of the samples (Figure 2A, bottom). To assess protein expression, Western blot analysis was performed using monoclonal antibodies against full-length PSA. The PSA protein was also expressed in all samples (Figure 2B). As the expression pattern of PSA was not concordant with that of sensitivity to 16F3, we speculated that 16F3 might recognize a distinct peptide derived from a separate protein that is antigenically close to the PSA-derived peptide. To examine this hypothesis, we investigated the CTL response against cells treated with PSA-specific siRNAs. The target sequences of three siRNAs were chosen outside of the PSA variant coding regions using BLOCK-iT™ RNAi Designer (Invitrogen). Because two pancreatic cancer cell lines, KP-3 and MIA PaCa-2, were able to accept siRNA efficiently, these cells were used in these experiments. The expression of the PSA protein was reduced in the cells treated with siRNAs specific for PSA (Figure 2C), which led to much lower capacities to stimulate 16F3 than with a negative control siRNA (Figure 2D), indicating that 16F3-mediated IFN-γ production occurs through recognition of PSA.

### The Processing of the PSA Epitope with the MHC Class I Involves the Vacuolar Pathway

**Figure 3:**
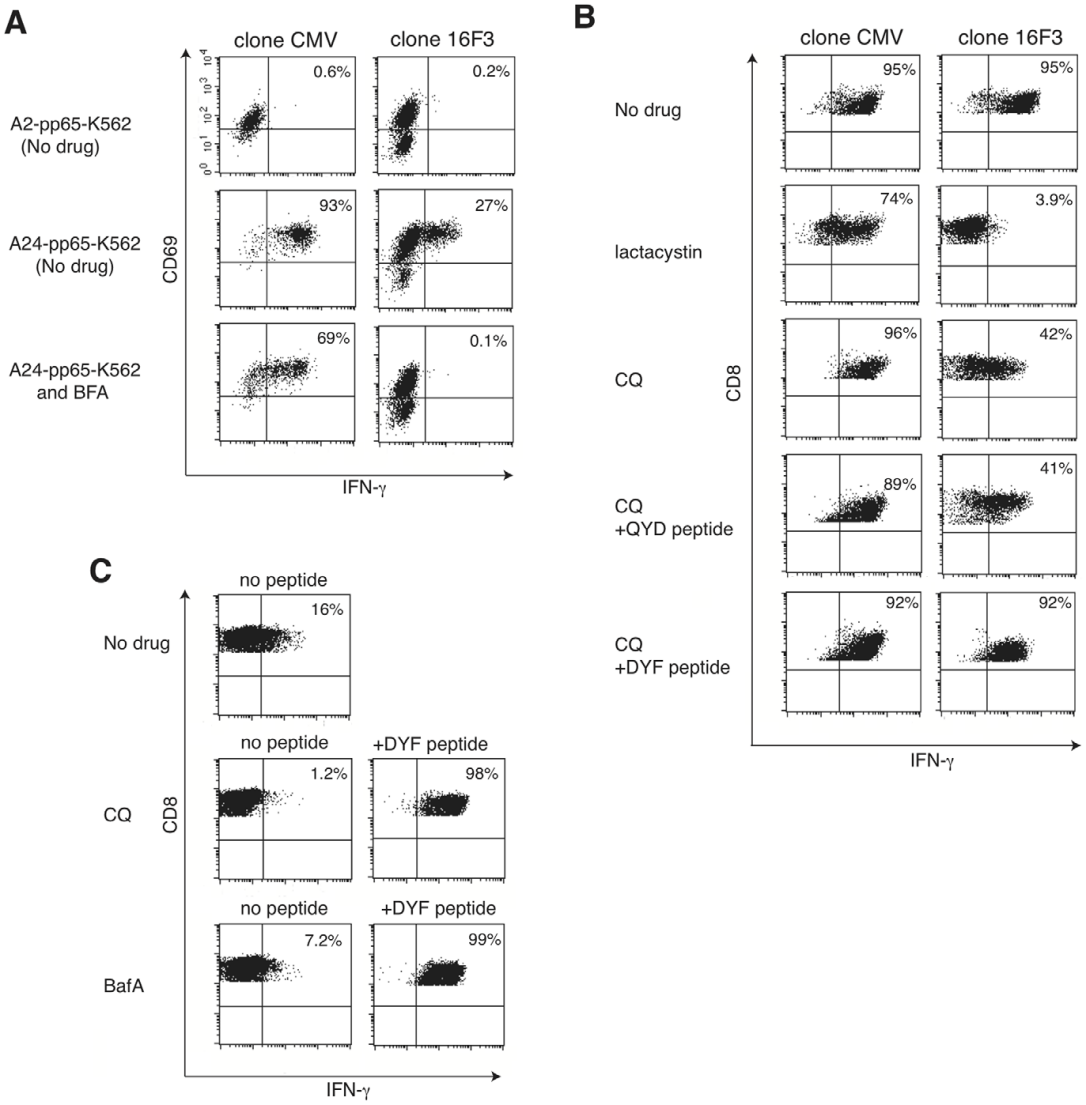
The epitope is presented and processed through a vacuolar pathway. A, K562 cells expressing both HLA-A2 or A24 and CMV pp65 (A2-pp65-K562 or A24-pp65-K562) were acid-stripped and incubated at 37°C for 9 h in the presence or absence of BFA. Then, the cells were co-cultured with either 16F3 or an HLA-A24-restricted CMV pp65-specific CTL clone for an additional 5 h. BFA was also added during the co-culture. After fixation and permeabilization, the cells were stained for CD3, CD8, CD69 and IFN-γ. CD3^+^ and CD8^+^ T cells were gated and analyzed using a flow cytometer. The frequency of IFN-γ producing cells is shown as the percentage of the total CD3^+^ CD8^+^ T cells. B-C, IFN-γ secretions of clones for 4 h after stimulation with A24-pp65-K562 cells (B) or KP-3 cells (C) treated with acid buffer for peptide stripping and/or inhibitors for 14 h was detected using an IFN-γ catch assay. 7-AAD^−^ alive CD8^+^ T cells were gated and analyzed using a flow cytometer. The frequency of IFN-γ secreting cells is shown as the percentage of the total alive CD8^+^ T cells. Whereas irreversibly acting lactacystin was removed during co-culture period (B), CQ and Baf A were retained in the media (B, C) because of their reversible nature. To exclude the possibility that CQ and Baf A could be inhibitory for 16F3 to produce IFN-γ, cognate or irrelevant peptides were added at concentrations of 1 µg/ml (B, C) and the T-cell response was examined.

Next, we addressed the mechanisms underlying the epitope generation using specific inhibitors, as no difference in the mRNA expression of TAP1, TAP2, LMP2 or LMP7 was evident between 16F3-sensitive and 16F3-insensitive cells (data not shown). For this purpose, we prepared a CMV pp65-expressing aAPC [12]. Fixed APCs are typically used to examine the effects of drugs on antigen-processing mechanisms [13]. However, 16F3 did not recognize paraformaldehyde- or glutaraldehyde-fixed A24-K562 cells (Figure S3A), and further treatment of quenching with glycine did not recover the epitope recognition (Figure S3B). Therefore, IFN-γ production was measured in the presence of reversible inhibitors, such as BFA, CQ and BafA, to avoid the restoration of the epitope following inhibitor removal during co-culture with T-cells (Figure 3A–C). IFN-γ production was detected via intracellular staining with BFA, as this drug causes the accumulation of IFN-γ within the cytosol (Figure 3A), and via secretion assays with other inhibitors (Figure 3B, 3C). IFN-γ production by 16F3 was decreased after treatment with both a proteasome inhibitor, lactacystin and with BFA, inhibiting the endoplasmic reticulum to Golgi transport (Figure 3A, 3B). This effect was also observed for another HLA-A24-restricted CTL clone specific to CMV pp65 [14], indicating the involvement of the classical MHC class I presentation pathway. Unexpectedly, CQ, an inhibitor of acidification inside the endosome/lysosome, decreased epitope processing from PSA, but not from the CMV protein (Figure 3B). In addition, KP-3 cells treated with either CQ or BafA, another acidification inhibitor, decreased PSA epitope presentation (Figure 3C). CQ and BafA did not affect T-cell function during co-culture, as demonstrated by intact IFN-γ secretion by cognate peptide-pulsed target cells in the presence of the inhibitors (Figure 3B, 3C). These data indicate that a vacuolar pathway mediates the processing of the PSA epitope, contrasting with the classical MHC class I pathway for the CMV epitope, although both utilize the same HLA-A24 molecules for presentation.

Two examples have been reported with regard to MHC class I molecules loading epitopes via CQ-sensitive vacuolar pathways. One example indicates that recycled MHC class I molecules, as with to MHC class II, can assemble with peptides in the acidic environment of the late endosome [15]. The other example involves the intracellular trafficking of peptides to the endosome mediated by specific signals within the cytoplasmic domain of class I molecules [16]. To examine the above possibilities, we generated mutant HLA-A24 molecules that were reported to lack any capacity for internalization and endosomal/lysosomal trafficking [16]–[18]. HLA-A24-YA contains a single point mutation replacing a tyrosine of exon 6 with alanine, and HLA-A24-YAΔ7 has a deletion of exon 7 and the same tyrosine substitution (Figure S4A). CMV pp65-expressing K562 was stably transduced with lentiviral vectors expressing those mutated HLA-A24 forms. All of the constructs similarly expressed HLA on the cell surfaces (Figure S4B). Both HLA mutants were recognized via either 16F3 or CMV clones as efficiently as wild type HLA-A24 (Figure S4C). In addition, primaquine, an inhibitor of the recycling of HLA class I from the endosome to the cell surface [19], did not inhibit PSA epitope presentation (data not shown). These data indicate that both MHC class I molecule recycling and an endosomal trafficking pathway of MHC class I molecules are irrelevant to the generation of the epitope.

### Constitutively Active Autophagosomes are Indispensable for the PSA Epitope Generation

**Figure 4:**
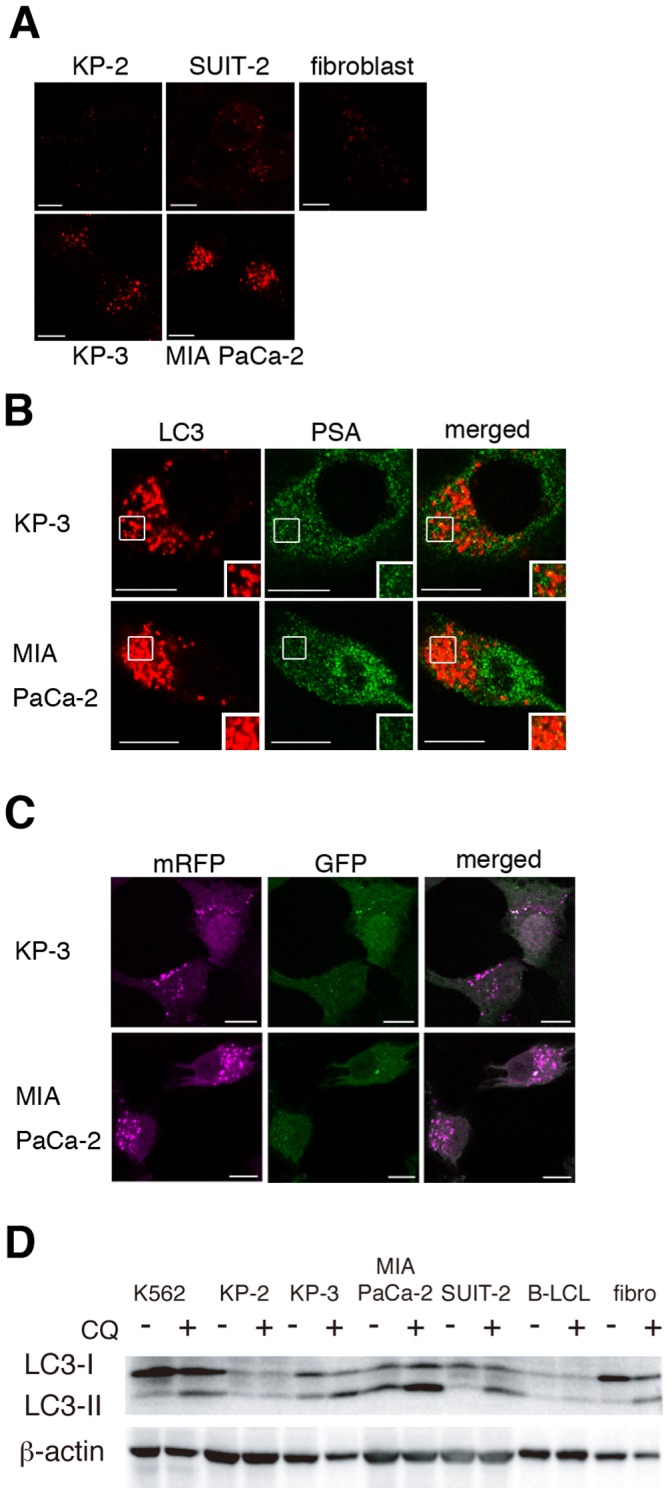
Constitutively active autophagy is involved in the 16F3 epitope processing of cancer cells. A, An immunofluorescence assay was performed to examine the expression of endogenous LC3. B, Double staining for endogenous LC3 and PSA was performed. Cells were cultured with BafA for 4 h, and then immunocytochemistry was performed. A yellow signal indicates colocalization. C, KP-3 and MIA PaCa-2 cells were transfected with plasmids expressing an mRFP-GFP-LC3 tandem-tagged fluorescent protein. Forty hours after transfection, the cells were fixed and analyzed via microscopy. A white signal indicates colocalization. The bars indicate 10 µm (A–C). D, The status of autophagic flux was measured via the LC3-II expression level. Cells were cultured with or without CQ for 2 h, and then the cell lysates were subjected to Western blot analysis for LC3.

As CQ and BafA are known to inhibit lysosomal function, we evaluated the level of autophagy using an immunofluorescence assay to detect the autophagosomal marker LC3. KP-3 and MIA PaCa-2 cells, which process and present the PSA epitope, had strong punctate LC3 staining, whereas SUIT-2, KP-2 and normal fibroblast cells that were not sensitive to CTL recognition showed weak staining (Figure 4A). Moreover, colocalization of autophagosomes and PSA was observed with BafA treatment for 4 h (Figure 4B), indicating that the protein might become a substrate of autophagic digestion in KP-3 and MIA PaCa-2 cells. To examine whether autophagic puncta could progress to autolysosomes in these cells, the autophagic flux was measured using a difference in the sensitivity of GFP and mRFP to the lysosomal environment [20]. When mRFP-GFP-LC3 tandem-tagged fluorescent protein entered the autolysosome from the autophagosome, the GFP fluorescence signals would be diminished, but the mRFP would remain fluorescent. KP-3 and MIA PaCa-2 cells transfected with the plasmid had many mRFP puncta in autolysosomes, with few colocalized signals of GFP/mRFP in autophagosomes (Figure 4C), implying that these cells have a high autophagic flux state and that the LC3 puncta were not mere aggregates. Furthermore, we measured the flux status by evaluating an autophagosomal marker, LC3-II, which digests itself in the autolysosome. The status of the autophagosome is accurately represented by differences in the amount of LC3-II between samples in the presence and absence of lysosomal inhibitors, such as CQ, that block the degradation of the autolysosome [21]. KP-2 cells and B-LCLs expressed LC3-II at low levels, and changes in the LC3-II band were limited on the addition of CQ (Figure 4D). However, LC3-II levels were markedly increased in K562, KP-3 and MIA PaCa-2 cells that were recognized efficiently by 16F3 (Figure 4D). Fibroblasts and SUIT-2 cells had low or moderate autophagic flux. These data suggest that the epitope presentation requires constitutively active autophagosomes and autophagic flux.

### PSA Epitope Processing and Presentation in Pancreatic Cancer Cells Engages the Autophagic Pathway

**Figure 5:**
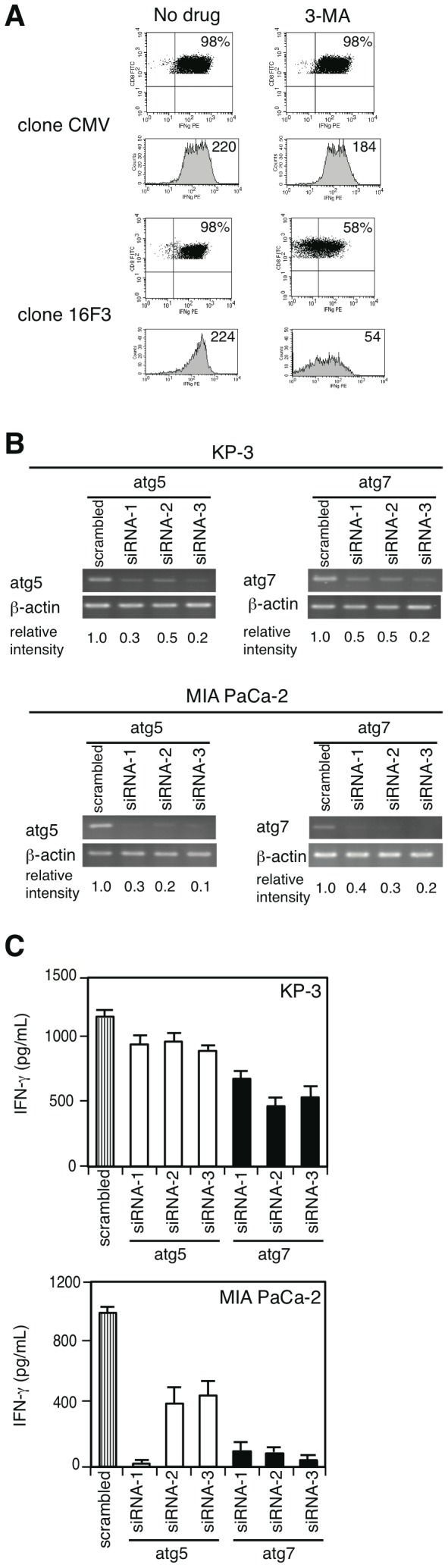
Autophagy is involved in the 16F3 epitope processing in cancer cells. A, K562 cells expressing both HLA-A2 or A24 and CMV pp65 were treated with an acid buffer for peptide stripping and incubated for 14 h in the presence or absence of 3-MA. Next, the cells were co-cultured with each clone for 4 h for IFN-γ secretion detected via the IFN-γ catch assay. The frequency of IFN-γ-secreting cells is shown as the percentage of the total living CD8^+^ T cells. 3-MA was not added during the co-culture period. In the lower panels, histograms of the IFN-γ signal and their mean fluorescence intensity are shown. B, RT-PCR analysis of scrambled, atg5- or atg7-specific siRNA-treated cells performed 70 h after transfection. The intensities of the bands were calculated using the ImageJ software. C, The CTL response against KP-3 and MIA PaCa-2 cells transfected with scrambled, atg5- or atg7-specific siRNA for 70 h was examined using an IFN-γ ELISA. Three different siRNA targets were chosen for each autophagy-associated gene. The results are the means ± SD of triplicates. Similar results were obtained in three separate experiments.

Recently, autophagy has been shown to deliver cytosolic proteins continuously to endosomes/lysosomes for antigen loading onto MHC class II molecules [22]. To address the involvement of an active autophagic pathway in antigen presentation onto MHC class I molecules, we first treated CMV pp65-expressing A24-K562 cells with 3-MA, a commonly used inhibitor of autophagy. We observed a decrease in IFN-γ production by 16F3 (Figure 5A) but not by CMV-specific CTLs. To confirm that autophagic processing is critical for epitope generation, siRNAs specific for two atgs, atg5 and atg7, were independently transfected into KP-3 and MIA PaCa-2 cells to suppress the protein expression and resultant autophagosome formation [21], [23]. After the siRNA treatment, atg5 and atg7 mRNA expression levels were reduced in KP-3 and MIA PaCa-2 (Figure 5B). Knockdown of each gene in both cells resulted in a decreased IFN-γ production by 16F3 (Figure 5C). HLA-A24 expression was not significantly changed on the surfaces of either 3-MA- or siRNA-treated cells compared with the control (Figure S5, S6). These results suggest that constitutively active autophagosomes play a significant role in epitope generation with MHC class I in cancer cells that have an elevated autophagic activity.

### Degradation of Full-length PSA Protein is Inhibited by CQ but Not by Lactacystin

**Figure 6:**
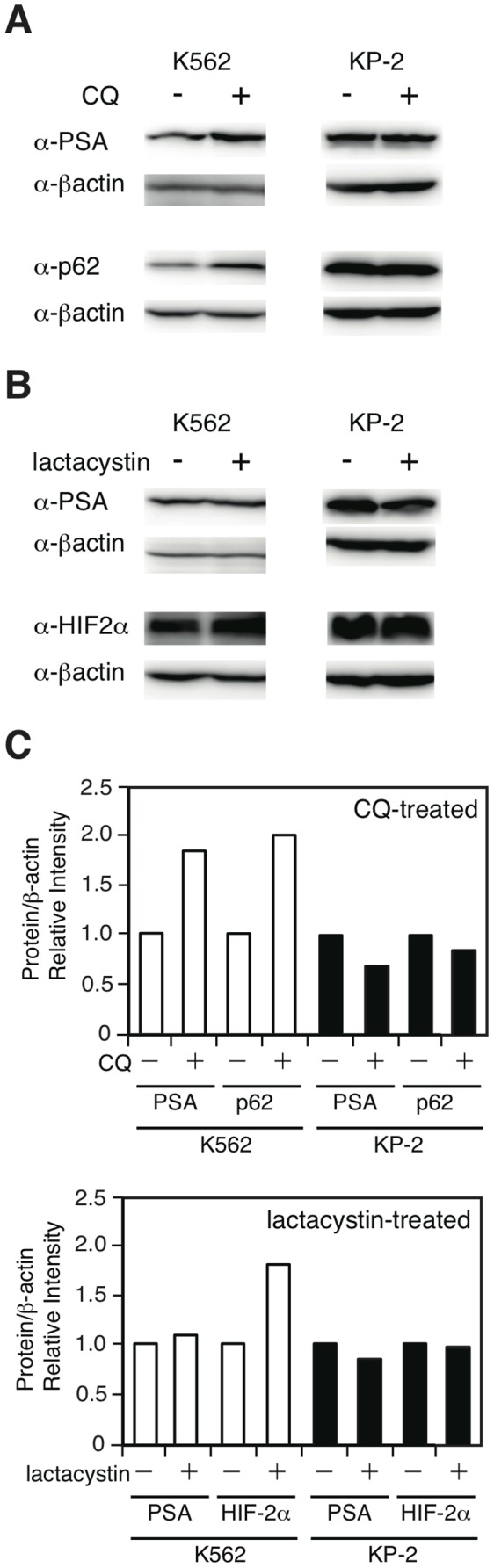
Degradation of full-length PSA protein is inhibited by CQ but not by lactacystin. A, Cells were cultured with or without CQ (50 µM) overnight, and then the cell lysates were subjected to Western blot analysis for PSA and p62 as a positive control for autophagic digestion. B, Cells were cultured with or without lactacystin (5 µM) overnight, and the cell lysates were subjected to Western blot analysis for PSA and HIF-2α as a positive control for proteasomal digestion. C, The intensities of the bands were calculated using the ImageJ software.

To provide an insight into the hierarchy of autophagy and proteasomal digestion to process PSA, K562 cells were treated with CQ, and the expression levels of the full-length PSA protein were examined by Western blot analysis. As shown in Figure 6A and C, the level of the PSA protein increased in K562 following CQ treatment. The increase was not observed in KP-2 cells with less autophagy. The increase in p62 protein, an efficient substrate of autophagy [24], was comparable in K562. Next, these cells were treated with lactacystin, and the levels of PSA along with HIF-2α protein, a substrate of proteasomal digestion [25], were examined. The level of PSA in K562 cells was not changed, whereas that of HIF-2α was increased (Figure 6B, 6C).

### Autophagy-induced Fibroblast Cells can Not Generate the PSA Epitope

**Figure 7:**
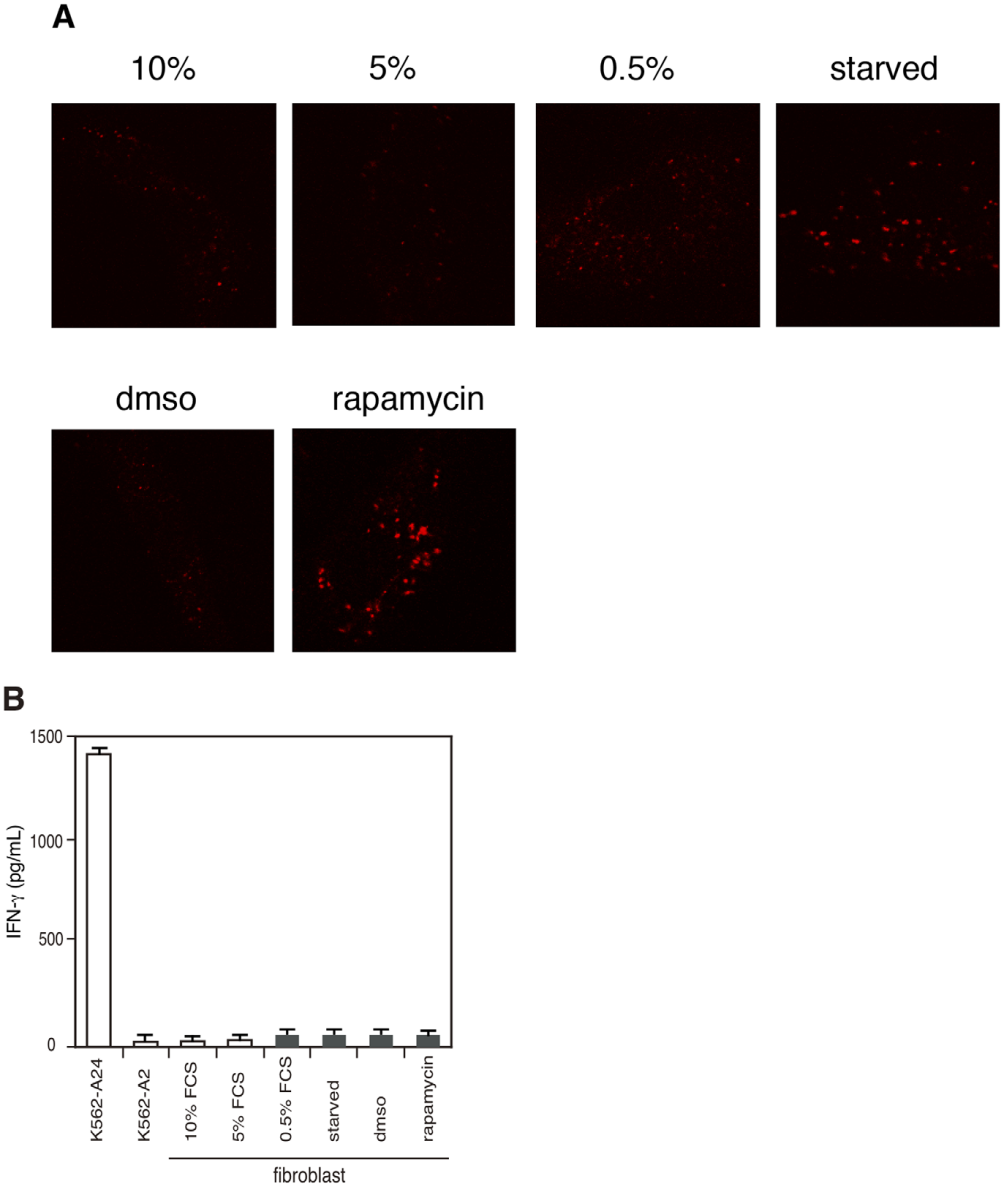
Induced autophagy does not result in PSA epitope presentation in fibrobast cells. A. Immunofluorescence assays for endogenous LC3 in fibroblast cells after low nutrient culture or rapamycin treatment. In low nutrient conditions, fibroblast cells were cultured in medium supplemented with 10%, 5%, 0,5% FCS or in Hank’s Balanced Salt Solution (starved). B, CTL response to autophagy-induced fibroblast cells treated with low nutrient culture conditions or rapamycin. Target cells were treated with low nutrient culture conditions or rapamycin for 4h, washed twice and cultured with CTL overnight. Next day, supernatants were harvested and IFN-γ measured by ELISA. K562-A24 cells and K562-A2 cells were used as positive and negative control, respectively. The results show means ± SD of triplicates.

Finally, we tested whether rapamycin, an m-TOR inhibitor being well documented as an inducer of autophagy, or low nutrient culture conditions could induce normal fibroblast cells to create the epitope. As shown in Figure 7A, autophagosomes increased by the drug treatment and by low nutrient culture conditions. However, these cells ere not recognized by 16F3 (Figure 7B). The same treatments did not induce B-LCLs to present the epitope to 16F3 (data not shown). These data demonstrate that, in normal cells, induced autophagy can not create the epitope, suggesting that constitutively active autophagy in cancerous cells is required for the epitope creation.

## Discussion

The present study demonstrated, using cDNA expression cloning, that a variant PSA was an antigen recognized by 16F3, although full-length PSA also stimulated 16F3. Remarkably, despite the ubiquitous expression of both the variant and full-length forms, the 16F3 CTL was found preferentially to recognize cancer cells but not normal cells. As other examples of this phenomenon, transcripts of p15 and cDNA31.2 are ubiquitously expressed, yet relevant CTLs only recognize tumor cells and not normal cells, including B-LCLs [26], [27]. Herein, we demonstrate a new mechanism of antigen processing and presentation that emphasizes the differences between cancer and normal cells with respect to ubiquitously expressed proteins. Based on the present results with specific inhibitory chemicals and siRNA targeting autophagy, and monitoring the results of autophagic activities, epitope liberation is caused by differences in the autophagic status, and therefore, a ubiquitously expressed protein, PSA, mimics tumor-associated antigens through a unique mechanism involving autophagy.

Our data indicate that both autophagosomes and proteasomes are necessary for the processing of the epitope peptide from PSA. Taking into consideration that lactacystin considerably decreases PSA epitope presentation to CTLs (Figure 3B) without changing the level of full-length PSA protein (Figure 6C), it is likely that proteasomes cleave smaller fragments of PSA to create the epitope. We speculate that these data suggest that PSA is degraded through the autophagosome-lysosome pathway prior to digestion in the proteasome.

Autophagy plays an important role in tumor initiation and progression in contrasting manners. Cancer cells use autophagy to survive and propagate in hypoxic and low-nutrient microenvironments, whereas autophagy is suppressive of tumor initiation because of the clearance of mutations. Increased autophagy has been shown in late-stage colon cancer [28], breast cancer [29], melanoma [30], hepatoma [31] and malignant glioma [32]. Additionally, for pancreatic cancer, autophagy correlates with a poor outcome [33] and is required for tumor growth [34]. Moreover, autophagy is associated with mutated K-ras-induced malignant transformation [35], [36]. Taken together, most pancreatic cancer cells with a K-ras mutation at codon 12 should have constitutively active autophagy. We demonstrated that an autophagosomal marker LC3 was strongly expressed in KP-3 and MIA Paca-2 cells. These pancreatic ductal cancer cell lines have a K-ras mutation at codon 12, whereas autophagosome is basal level in KP-2 cells having no K-ras mutation [37]. Remarkably, rapamycin treatment and starvation did not induce normal fibroblast cells to create the epitope, although autophagosome was observed. Our data suggest that aberrant increases in autophagosomes in association with K-ras mutation create distinct peptide repertoires displayed on the MHC class I molecules of cancer cells, potentially evoking CTL responses while sparing normal cell damage.

In this study, a long 12-mer epitope antigen derived from PSA was identified, consistent with an earlier investigation that revealed an unusually long epitope presented on MHC class I [38]. Not only viral antigens but also tumor antigens are naturally processed into longer epitopes, and higher immunogenicities are indicated even in cancer patients [39]. The structures of the long peptides on MHC have been characterized, and bulging conformations have been observed [40]. In particular, HLA-B alleles preferentially bind to peptides over 11 residues in length, rather than to other HLA alleles. In this study, we demonstrated that HLA-A24-binding 12-mer peptides were created by autophagy. The epitope DYFNVPYPLKI has anchor residues for HLA-A24, such as a tyrosine at the second position and an isoleucine at the carboxyl-terminus, assuming a bulged formation in the middle of the epitope peptides. The unique processing pathway through autophagy may generate the long epitope peptide.

Concerning adaptive immunity, the report of autophagy-enhancing endogenous presentation on MHC class I is clearly of interest [13]. HSV type 1 (HSV-1) was found to trigger a vacuolar response that increased the presentation of a peptide derived from HSV-1 glycoprotein B to CTL on MHC class I. In HSV-1-infected murine macrophages, LC3-positive four-layered membrane structures emerged from nuclear envelopes and were uniquely found in “classical” autophagosomes, consisting of double-layered structures formed in the cytosol of uninfected macrophages. Furthermore, virus-induced four-layer forms appear to function similarly to autophagosomes and participate in the presentation of HSV-1 glycoprotein B on MHC class I. The difference in our data is that our data support a constitutively active autophagic pathway that contributes to the presentation of a self antigen by MHC class I on cancer cells.

In conclusion, the present investigation provides information on the contribution of autophagy to MHC class I processing and presentation in cancer cells but not in normal cells. Importantly, PSA displays differential susceptibilities to antigen presentation between cancer and normal cells, although it is expressed ubiquitously in both cases. Epitope creation from PSA unambiguously requires autophagic pathways, but elucidation of the more detailed molecular mechanisms and essential proteases awaits further studies. Furthermore, to what extent autophagy may participate in the generation of MHC class I-restricted epitopes remains unknown. The comprehensive analysis of peptides bound to MHC class I molecules on autophagy-knocked down cancer cells using mass spectrometry may answer the question, promoting a better understanding of cancer immunity and adding information for effective cancer immunotherapy.

## Materials and Methods

### Ethics Statement

The study design and purpose, after prior approval by the Institutional Review Board of the Aichi Cancer Center were fully explained, and written consent was obtained from healthy blood donors.

### Cell Lines

The human pancreatic cancer cell lines were purchased from the Japanese Collection of Research Bioresources. HLA-A24-positive normal human bronchial epithelial cells were cultured according to the supplier’s recommendations (CC2540, Takara, Ohtsu, Japan). An HLA-A24-positive dermal fibroblast line and TAP-deficient T2-A24 cells were cultured as previously described [14]. The retroviral transduction of CMV 65 kDa phosphoprotein (pp65) was performed as previously described [12].

### Construction of Artificial Antigen-presenting Cells (aAPCs)

Vectors carrying HLA-A*02∶06, -A*24∶02, mutated HLA-A*24∶02, human CD86 and human 4-1BBL genes under the EF-1α promoter were constructed (CSII-EF-MCS) [41]. Lentiviral transduction was performed with similar retroviral methods [12].

### CTL Induction

Naïve CD8^+^ T cells were negatively isolated from PBMCs of A24-positive donors using the CD8^+^ T cell isolation kit II and anti-CD45RO microbeads (Miltenyi Biotec, Bergisch Gladbach, Germany). The isolated cells were more than 95% pure CD45RO^−^ CD8^+^ populations. These CD8^+^ T cells (1×10^6^ cells/well) were cocultured with irradiated (100 Gy) aAPCs (1×10^5^ cells/well) in 2 mL X-VIVO20 (CAMBREX, East Rutherford, NJ) supplemented with 5% human AB serum (MP Biomedicals, Solon, OH) in wells of a 12-well plate in the presence of 10 ng/mL IL-12 (Peprotech, Rocky Hill, NJ)). On day 3, 10 ng/mL IL-7 (Peprotech) and IL-15 (Peprotech) were added. Every 3 days, half the medium was exchanged for fresh medium containing 10 ng/mL IL-15. On day 12, the T cells were restimulated with γ-irradiated aAPCs. One day thereafter, IL-2 (Primmune, Kobe, Japan) was added, to a final concentration of 20 U/mL. To establish T cell clones, a limiting dilution of the polyclonal CTL was performed as previously described [14].

### Detection of IFN-γ Producing CD8^+^ T Cells

An IFN-γ secretion assay to detect IFN-γ release with a PE-labeled antibody was performed using a kit (Miltenyi Biotec) and following the manufacturer’s recommendations. In inhibition assays, after an acid-stripping treatment, as described previously [42], the stimulators were incubated with drugs at the following concentrations overnight: brefeldin A (BFA, Sigma-Aldrich, St. Louis, MO), 10 µg/mL; lactacystin (Calbiochem, Darmstadt, Germany), 20 µM; chloroquine (CQ, Sigma-Aldrich), 100 µM; bafilomycin A1 (BafA, Sigma-Aldrich), 250 nM; and 3-methyladenine (3-MA, Sigma-Aldrich), 10 mM. BFA, CQ and BafA were added during co-culture with T-cells, while lactacystin and 3-MA were not. After staining, flow cytometric analysis of the stained cells was performed using a FACSCalibur (BD Biosciences, Franklin Lakes, NJ), and the data were analyzed with the help of CellQuest Pro software (BD Biosciences). Synthetic epitope peptides (Greiner, Frickenhausen, Germany) were added at concentration of 1 µg/mL to confirm intact IFN-γ production by T-cells in the presence of the drugs. In certain experiments, intracellular cytokine assessment using flow cytometry was performed instead of an IFN-γ secretion assay, as previously described [43].

### Construction of a cDNA Library and Expression Screening by ELISA

The preparation of a cDNA library and expression screening were performed as described previously [44]. After overnight culture with 16F3, IFN-γ in the supernatant was measured by ELISA.

### MHC Stabilization Assay

An MHC stabilization assay was performed to test the peptide for HLA-A24 binding efficiency using T2-A24 cells, as described earlier [14]. Briefly, T2-A24 cells were incubated with RPMI 1640 containing 0.1% FCS and each of the peptides at a concentration of 10 µg/mL at 26°C for 16 h, followed by incubation at 37°C for 3 h. After incubation, surface HLA-A24 molecules were stained with the anti-A24 mAb and anti-mouse FITC-conjugated Ab. HLA-A24 expression was measured using a FACSCalibur (BD Biosciences), and the mean fluorescence intensity was analyzed using the Cellquest Pro software (BD Biosciences).

### IFN-γ ELISA for the Evaluation of Antigen Recognition by 16F3 with Fixed Stimulators

K562 cells expressing both HLA-A24 and CMV pp65 were fixed by various methods previously described [45], [46] with slight modification as follows: fixation with 0.2% paraformaldehyde for 10 min, fixation with 0.008% glutaraldehyde for 3 min or fixation with 0.05% glutaraldehyde for 30 sec. After fixation, cells were washed with RPMI 1640 medium plus 10% FCS five times. For quenching, the cells were further washed with PBS containing 10 mM glycine two times. Following overnight co-culture of T-cell and the stimulators, the expression of IFN-γ in the cell culture supernatant was determined using an ELISA.

### Plasmid Construction of HLA-A24 Mutants

A plasmid expressing HLA-A24, pcDNA3.1(+)/HLA-A24, was constructed as previously described [12]. To construct an HLA-A24-YA mutant [16] containing a single point mutation substituting an alanine residue for the tyrosine residue of exon 6, overlapping PCR [47] was performed, using pcDNA3.1(+)/HLA-A24 containing a T7 promoter primer binding site and BGH reverse priming site as a template. The following primers were used: sense primer for the T7 promoter primer-binding site, 5′-TAATACGACTCACTATAGGG-3′ (T7pp primer); internal antisense primer, 5′-CAGCCTGAGAGGCGCTCCCTCCTTTTCTATCTGAG-3′; an internal sense primer, 5′-AAGGAGGGAGCGCCTCTCAGGCTGCAAGCAGTGA-3′; an antisense primer for the BGH reverse priming site (BGHrp primer), 5′- TAGAAGGCACAGTCGAGG-3′. The next PCR was performed with the first PCR products as templates, using the T7pp primer and the BGHrp primer, and produced the fusion product. To construct the HLA-A24-YA-Δ7 mutant [16] containing a complete deletion of exon 7 and a single point mutation substituting an alanine residue for the tyrosine residue of exon 6, PCR was performed with pcDNA3.1(+)/HLA-A24 as a template using the T7pp primer and an antisense primer, 5′-AAGCGGCCGCTCACACTGCAGCCTGAGAGGCGCTCCCTCC-3′. The resultant PCR fragments were cloned into lentiviral expression vectors (CSII-EF-MCS). The cloned genes were sequenced to verify their identity.

### Immunofluorescence Microscopy

Cells on glass coverslips were fixed for 10 min with PBS containing 3% paraformaldehyde. After washing, the cells were permeabilized with 50 µg/mL digitonin (Sigma-Aldrich) in PBS for 5 min at room temperature. The cells were then washed and blocked with 3% BSA in PBS for 10 min. After probing with a rabbit Ab specific to human PSA (GeneTex, Irvine, CA) and a mouse mAb specific to human LC3 (4E12, MBL, Nagoya, Japan) for 1 h, secondary Abs conjugated to Alexa 488 and 594 (Invitrogen, Carlsbad, CA) were applied for 30 min. All of the immunochemical assays were performed at room temperature. The slides were mounted in ProLong Gold antifade reagent (Invitrogen). Confocal fluorescence images were obtained using a confocal laser-scanning microscope (LSM510 META; Carl Zeiss, Göttingen, Germany) with a Plan-Apochromat 63x/1.40- or 100x/1.40- numerical -aperture oil immersion lens and LSM Image Browser software (Carl Zeiss).

### Western Blot Analysis

Western blot analysis was performed as described previously [48]. A mouse mAb specific to human PSA (#2649C4a; Cosmo Bio, Tokyo, Japan), p62 (5F2; MBL) HIF2α (#NB100-132; NOVUS, Littleton, CO) or β-actin (Sigma-Aldrich), or a rabbit Ab specific to human LC3 (MBL) was used. All of the images were processed by Lumi Vision Pro 400EX (Aisin/Taitec, Inc., Kariya, Japan). The signal intensity was quantified with the ImageJ software.

### Transfection with Small Interfering RNAs (siRNAs)

SiRNAs specific for PSA, autophagy-related gene (atg)5 and atg7 were created using Stealth™ RNAi preparations and obtained from Invitrogen. The target sequences are as follows: PSA-siRNA-1, GGAGGAUUCUUAAUAUCCAGACUAA; PSA-siRNA-2, CAGCUCUGCCAUGCUGGAAAGUUUA; PSA-siNRA-3, CCAAACCUGGAGAAGGUCAUCUCGA; atg5-siRNA-1, UCGAGAUGUGUGGUUUGGACGAAUU; atg5-siRNA-2, AAGCAACUCUGGAUGGGAUUGCAAA; atg5-siRNA-3, CCUCAAAGAAGUUUGUCCUUCUGCU; atg7-siRNA-1, CAGAAGGAGUCACAGCUCUUCCUUA; atg7-siRNA-2, GAAAGCCAUGAUGUCGUCUUCCUAU; and atg7-siRNA-3, GGCCGUGGAAUUGAUGGUAUCUGUU. A scrambled siRNA (Invitrogen) was used for negative control. The KP-3 and MIA PaCa-2 cells (2×10^5^ cells/well) were transfected with each siRNA (100 nM) using Lipofectamine 2000 (Invitrogen) in 24-well plates. After 70 h, the cells were harvested and used in an ELISA, or the total RNA was extracted using the RNAeasy mini kit (Qiagen, Tokyo, Japan) with DNase I treatment, and reverse transcription was performed in 20-ul reactions containing oligo (dT)_20_ primers and 0.5 µg aliquots. The alignments of specific primer sets were as follows: PSA and PSA variant common forward, 5′-TCCATCTGAGGTTGATGAGAT-3′; PSA reverse, 5′-TCCACATAAATGAGGGGAAATC-3′; PSA variant reverse, 5′-GTCCCAGCCTGGATAGAGTG-3′; atg5 forward, 5′-TGGGATTGCAAAATGACAGA-3′; atg5 reverse, 5′-CACTGCAGAGGTGTTTCCAA-3′; atg7 forward, 5′-GAAACCAAAGCAGCAAGGAG-3′; and atg7 reverse, 5′-CATTCATCCGATCGTCACTG-3′. The PCR products were separated in 2.0% agarose and visualized with ethidium bromide staining.

## Supporting Information

Figure S1 **Identification of PSA variant cDNA encoding an epitope recognized by 16F3.** A, HLA-A24-expressing HEK293T (A24-293T) cells were transfected with each plasmid from a cDNA library constructed from mRNA extracted K562 cells. IFN-γ was measured by ELISA. Arrow indicates a well of 8G containing an antigenic plasmid. B, The cDNA clone was a variant PSA (NM_006310). The schematic drawing of full length (top) and variant (bottom) PSA is shown. The variant is an intronic polyadenylated mRNA ending with the exon 12 and a following intron. C-D, Identification of an epitope peptide recognized by 16F3. A24-293T cells were transfected with each plasmid encoding truncated fragments. The constructs shown as open boxes were recognized by 16F3, while that shown as filled boxes were not. Numbers indicate amino acid positions (C). Minigenes were cloned into pcDNA3.1(+) plasmid and transfected into A24-293T cells. The amino acid sequences were shown in one-letter code, and defined epitope is underlined (D**)**. E, MHC stabilization assay was executed without peptide or with either synthetic 12mer peptide, DYFNVPYPLPKI or control CMV peptide, QYDPVAALF. F, T2-A24 cells were pulsed with serial concentrations of either synthetic 12mer peptide, DYFNVPYPLPKI (diamond) or control CMV peptide, QYDPVAALF (circle). Release of IFN-γ was measured by ELISA. The results are the mean of triplicate values.
(TIF)

Figure S2 **Peptide-pulsed KP-2 and SUIT-2 cells were recognized by 16F3.** KP-3 (white), KP-2 (black) and SUIT-2 (vertical stripe) cells were pulsed with either synthetic 12mer peptide, DYFNVPYPLPKI (DYF) or control CMV peptide, QYDPVAALF (QYD) ate a concentration of 1 µM. Release of IFN-γ was measured by ELISA.
(TIF)

Figure S3 **Fixation of target cells abolish recognition by 16F3, which is not recovered by glycine quenching.** A–B, K562 cells transfected with CMV pp65 and HLA-A24 were used as stimulators for either 16F3 or an HLA-A24-restricted CMV pp65-specific CTL clone. A, Stimulators were fixed in three ways; 0.2% paraformaldehyde (PFA) for 10 min, 0.008% glutaraldehyde (GA) for 3 min, or 0.05% glutaraldehyde (GA) for 30 sec. After fixation, cells were washed 5 times with RPMI medium containing 10% FCS. B, Fixed and washed stimulators were further washed two times with PBS containing 10 mM glycine for quenching. The stimulators were cultured with each clone and IFN-γ in the supernatants was measured by ELISA. The data express mead of duplicates.
(TIF)

Figure S4 **The epitope is not presented by recycling HLA-A24 molecules.** A, The amino acid sequences of the cytoplasmic portion of the wild-type HLA-A24 (WT-A24) and two endosome recycling-compromised mutants, designated as A24-YA and A24-YAΔ7, are shown as a single letter code. S, signal sequence; TM, transmembrane domain; CY, cytoplasmic domain. B, Surface expression of HLA-A24 or its mutant molecules on lentiviral-transfected K562 cells was measured using a flow cytometer. C, K562 cells transfected with CMV pp65 accompanied by HLA-A02, HLA-A24 or its mutant were incubated with either 16F3 or an HLA-A24-restricted CMV pp65-specific CTL clone, and an IFN-γ ELISA was performed. The results are expressed as the means of triplicate experiments.
(TIF)

Figure S5 **Surface HLA expression of HLA-A24-expressing K562 cells treated with acid buffer and 3-methyladenine (3-MA).** The surface expression of the HLA class I molecule of HLA-A24-expressing K562 cells was examined with a flow cytometer after the cells were treated with acid buffer for peptide stripping and incubated with or without 3-MA for 14 h. The white and shaded areas show samples incubated with FITC-labeled anti-HLA class I and isotype control mAb, respectively. Stained cells were analyzed using a flow cytometer.
(TIF)

Figure S6 **The surface HLA expression of siRNA-transfected pancreatic carcinoma cells.** KP-3 (A) and MIA PaCa-2 (B) cells were transfected with scrambled, atg5, or atg7-specific siRNA for 70 h. The white and shaded areas show samples incubated with FITC-labeled anti-HLA class I and isotype control mAb, respectively. The stained cells were analyzed using a flow cytometer.
(TIF)
