# Supplementary figures and images for: Autophagy Creates a CTL Epitope That Mimics Tumor-Associated Antigens
Source: PLoS One. 2012 Oct 11;7(10):e47126. doi: 10.1371/journal.pone.0047126 (PMC3469533; doi:10.1371/journal.pone.0047126)

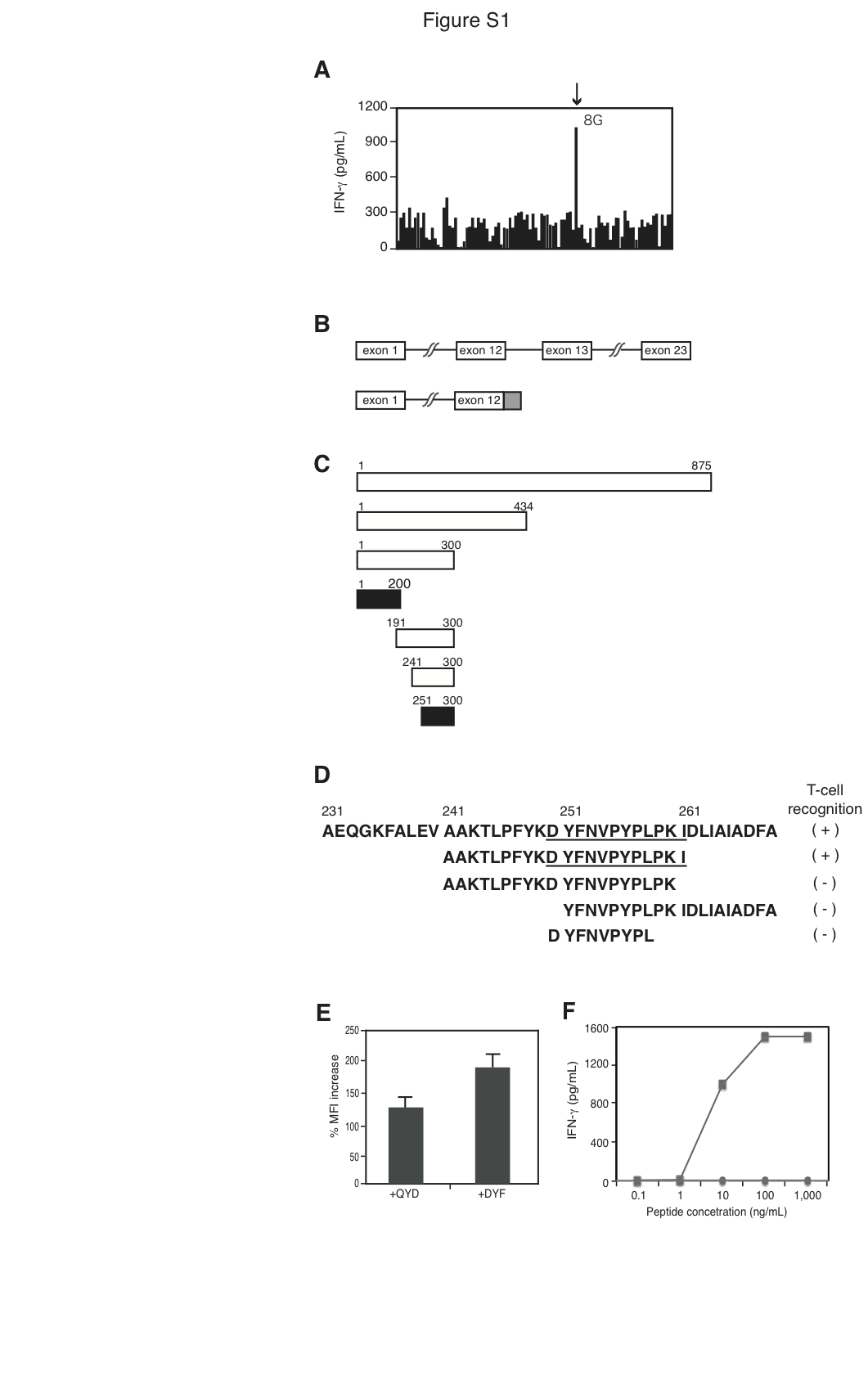

Supplement: Figure S1 — Identification of PSA variant cDNA encoding an epitope recognized by 16F3. A, HLA-A24-expressing HEK293T (A24-293T) cells were transfected with each plasmid from a cDNA library constructed from mRNA extracted K562 cells. IFN-γ was measured by ELISA. Arrow indicates a well of 8G containing an antigenic plasmid. B, The cDNA clone was a variant PSA (NM_006310). The schematic drawing of full length (top) and variant (bottom) PSA is shown. The variant is an intronic polyadenylated mRNA ending with the exon 12 and a following intron. C-D, Identification of an epitope peptide recognized by 16F3. A24-293T cells were transfected with each plasmid encoding truncated fragments. The constructs shown as open boxes were recognized by 16F3, while that shown as filled boxes were not. Numbers indicate amino acid positions (C). Minigenes were cloned into pcDNA3.1(+) plasmid and transfected into A24-293T cells. The amino acid sequences were shown in one-letter code, and defined epitope is underlined (D). E, MHC stabilization assay was executed without peptide or with either synthetic 12mer peptide, DYFNVPYPLPKI or control CMV peptide, QYDPVAALF. F, T2-A24 cells were pulsed with serial concentrations of either synthetic 12mer peptide, DYFNVPYPLPKI (diamond) or control CMV peptide, QYDPVAALF (circle). Release of IFN-γ was measured by ELISA. The results are the mean of triplicate values. (TIF) [file pone.0047126.s001.tif]

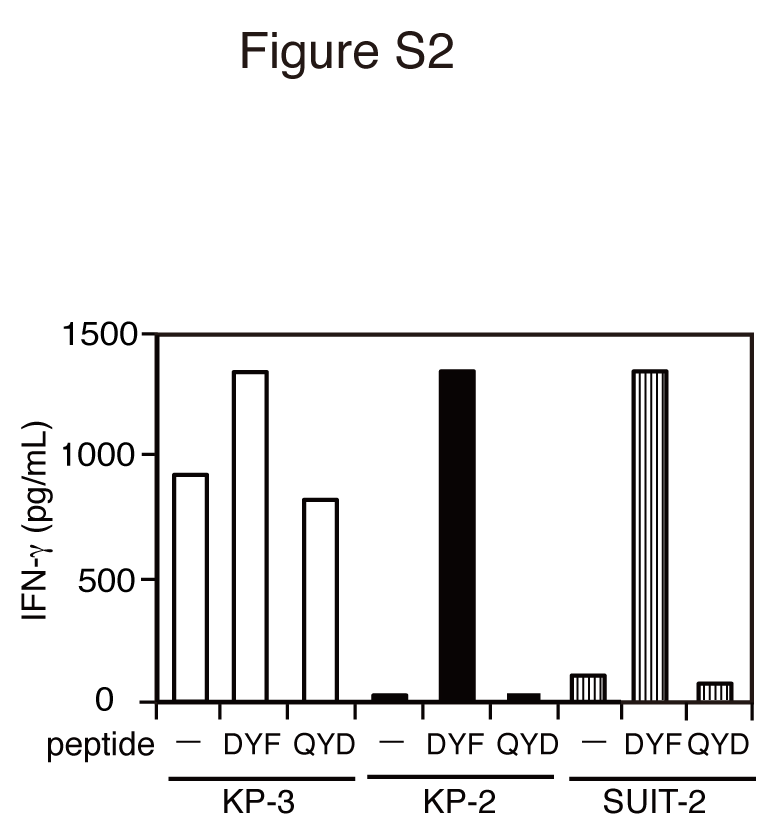

Supplement: Figure S2 — Peptide-pulsed KP-2 and SUIT-2 cells were recognized by 16F3. KP-3 (white), KP-2 (black) and SUIT-2 (vertical stripe) cells were pulsed with either synthetic 12mer peptide, DYFNVPYPLPKI (DYF) or control CMV peptide, QYDPVAALF (QYD) ate a concentration of 1 µM. Release of IFN-γ was measured by ELISA. (TIF) [file pone.0047126.s002.tif]

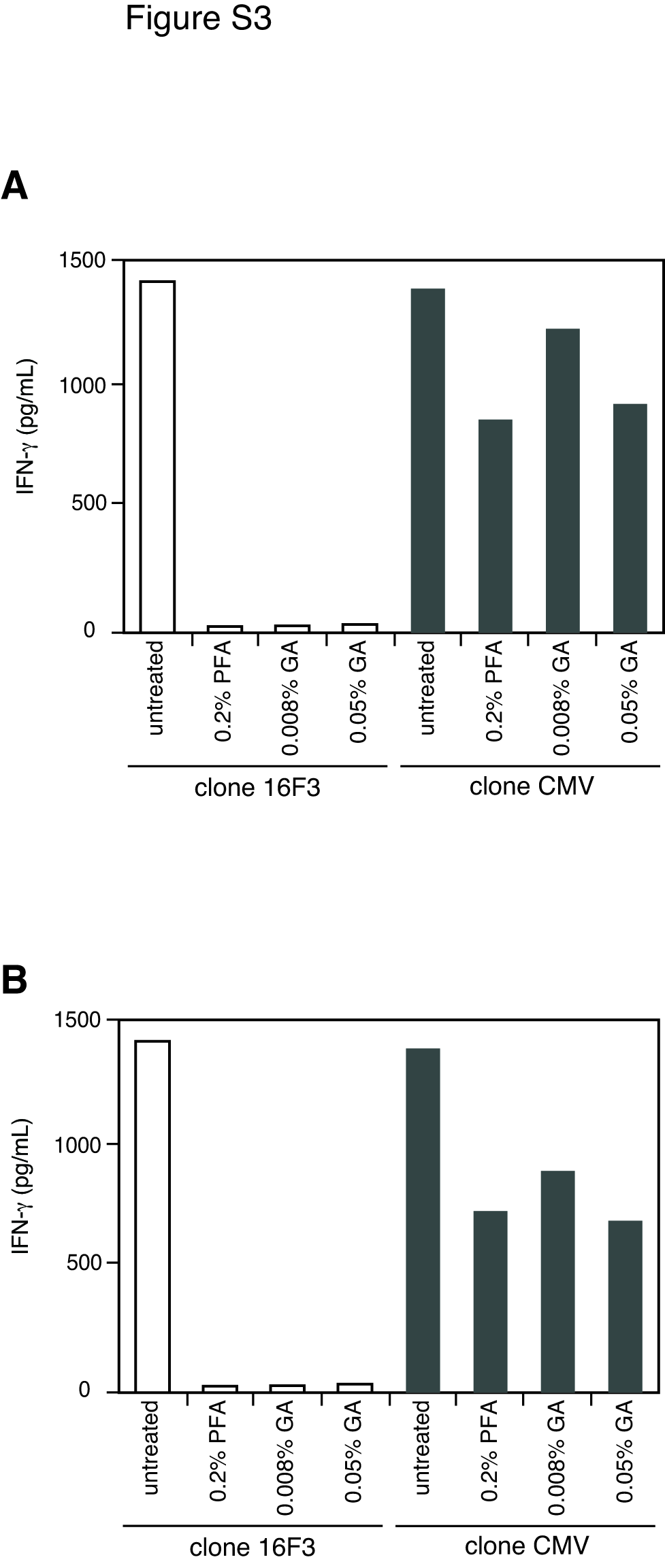

Supplement: Figure S3 — Fixation of target cells abolish recognition by 16F3, which is not recovered by glycine quenching. A–B, K562 cells transfected with CMV pp65 and HLA-A24 were used as stimulators for either 16F3 or an HLA-A24-restricted CMV pp65-specific CTL clone. A, Stimulators were fixed in three ways; 0.2% paraformaldehyde (PFA) for 10 min, 0.008% glutaraldehyde (GA) for 3 min, or 0.05% glutaraldehyde (GA) for 30 sec. After fixation, cells were washed 5 times with RPMI medium containing 10% FCS. B, Fixed and washed stimulators were further washed two times with PBS containing 10 mM glycine for quenching. The stimulators were cultured with each clone and IFN-γ in the supernatants was measured by ELISA. The data express mead of duplicates. (TIF) [file pone.0047126.s003.tif]

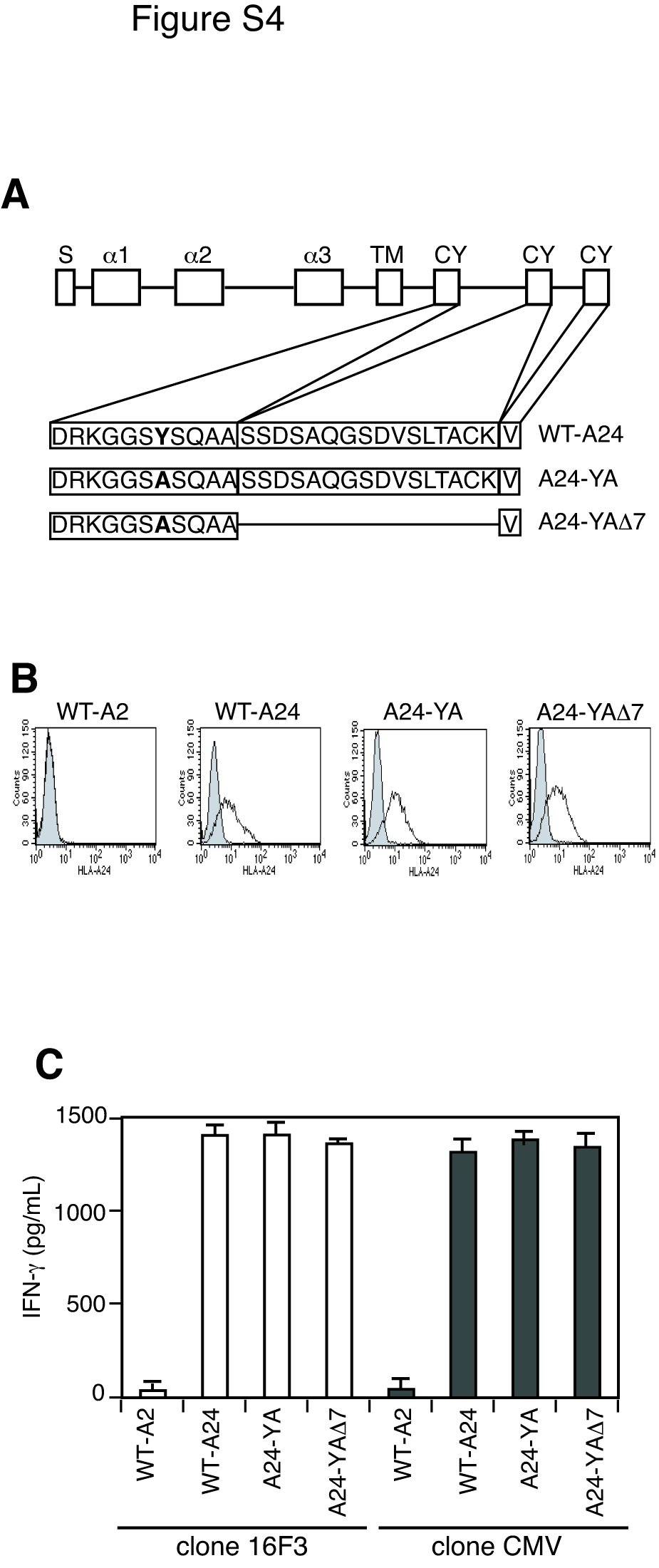

Supplement: Figure S4 — The epitope is not presented by recycling HLA-A24 molecules. A, The amino acid sequences of the cytoplasmic portion of the wild-type HLA-A24 (WT-A24) and two endosome recycling-compromised mutants, designated as A24-YA and A24-YAΔ7, are shown as a single letter code. S, signal sequence; TM, transmembrane domain; CY, cytoplasmic domain. B, Surface expression of HLA-A24 or its mutant molecules on lentiviral-transfected K562 cells was measured using a flow cytometer. C, K562 cells transfected with CMV pp65 accompanied by HLA-A02, HLA-A24 or its mutant were incubated with either 16F3 or an HLA-A24-restricted CMV pp65-specific CTL clone, and an IFN-γ ELISA was performed. The results are expressed as the means of triplicate experiments. (TIF) [file pone.0047126.s004.tif]

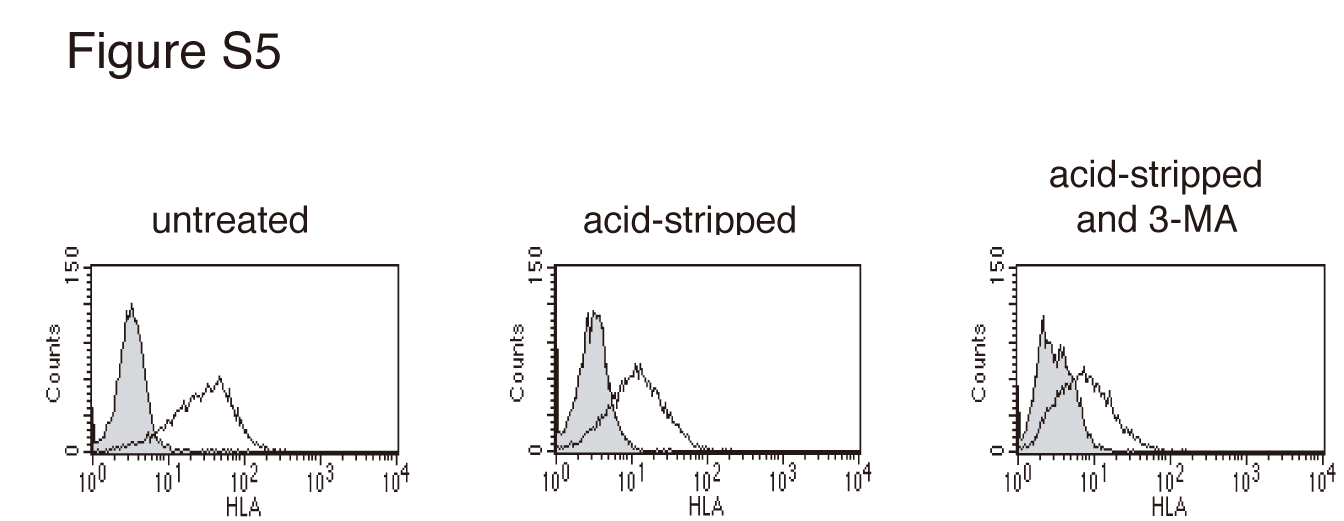

Supplement: Figure S5 — Surface HLA expression of HLA-A24-expressing K562 cells treated with acid buffer and 3-methyladenine (3-MA). The surface expression of the HLA class I molecule of HLA-A24-expressing K562 cells was examined with a flow cytometer after the cells were treated with acid buffer for peptide stripping and incubated with or without 3-MA for 14 h. The white and shaded areas show samples incubated with FITC-labeled anti-HLA class I and isotype control mAb, respectively. Stained cells were analyzed using a flow cytometer. (TIF) [file pone.0047126.s005.tif]

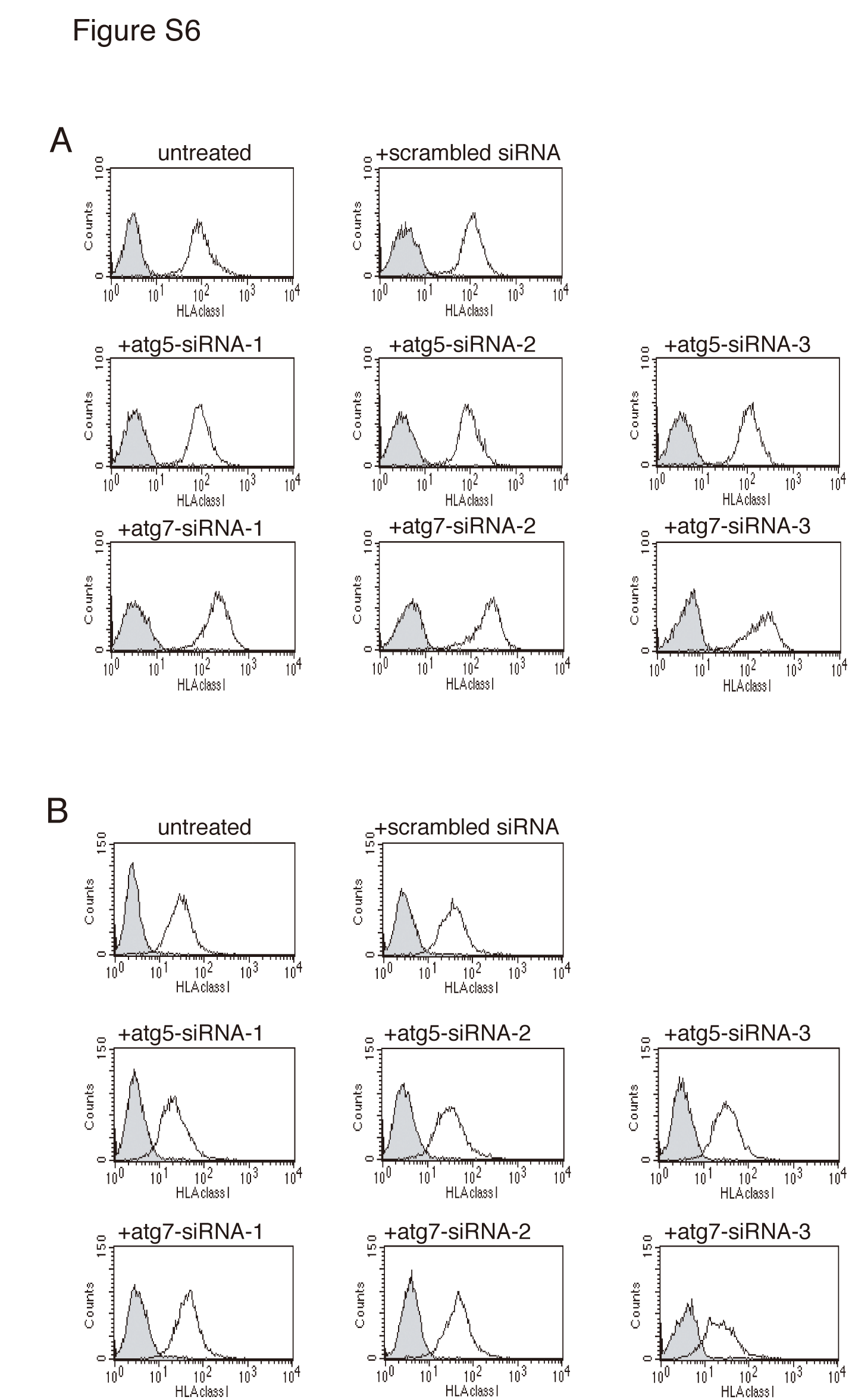

Supplement: Figure S6 — The surface HLA expression of siRNA-transfected pancreatic carcinoma cells. KP-3 (A) and MIA PaCa-2 (B) cells were transfected with scrambled, atg5, or atg7-specific siRNA for 70 h. The white and shaded areas show samples incubated with FITC-labeled anti-HLA class I and isotype control mAb, respectively. The stained cells were analyzed using a flow cytometer. (TIF) [file pone.0047126.s006.tif]
